# Psychiatry

**Published:** 1904-09-24

**Authors:** 


### PROGRESS IN MEDICINE AND SURGERY.

### PSYCHIATRY.

(*Concluded from page* 432.)

**General Paralysis and Crime.**—Dr. John Baker, of the Broadmoor Criminal Lunatic Asylum, states that during the prodromal stages of general paralysis failure and decay of the moral senses are the most important, and may sometimes show themselves in crime and petty outrages against order and property.[2] Acts of petty violence, indecent sexual acts (including rape), and boisterous drunkenness and disorderliness may be frequently exhibited in the earliest stages. The depressive or hypochondriacal form of early general paralysis is of graver import medicolegally than the expansive form, since graver crimes are liable to be committed by patients in the former state. On the other hand, the expansive, grandiose, and facile paretic usually is prone to commit petty acts of theft without any attempt at artifice or precaution. Of 62 general paralytics admitted to the Broadmoor Criminal Lunatic Asylum since it was opened, 54 were males and 8 were females. Every one of the cases showed the mental and physical features of the disease, and in every case of death post-mortem examination showed the characteristic pathological changes in the brain. First in medicolegal importance among them were the cases of homicide. Of these 9 were cases of murder, 6 of attempted murder, and 1 of aggravated assault, all the offenders being males. Of the 6 cases of murder committed in the depressive stage of onset of the disease, the offspring of the patients were the victims in every instance. "The feeling of bodily weakness, the gloomy affective tone, the sacrifice of children, are similarly seen in the lactational melancholia of nursing women, but in them suicidal feelings are invariably associated with the homicidal promptings, whereas the suicidal intent is rare in the melancholic variety of general paralysis." Four cases were instances of sexual offences, 3 being cases of rape, and 1 of carnal knowledge of a female. The 3 convicted of rape were found to be given to alcoholic habits, while also suffering from general paralysis; the fourth suffered from curious delusions of being possessed by a second (or "double") personality. The remaining cases included offences of the nature of arson (7 cases), and larceny and petty thieving (35 cases). Of the 7 cases charged with arson all belonged to the excited or grandiose type of general paralysis, and all denied the offence except one, who stated that he set fire to a hay-rick to clear the stack-yard. Of the cases of larceny, etc., 11 were of the exalted or grandiose type, 13 of the demented type, and 2 of the depressed type. The general paralytic who steals owing to his mental failure and incapacity, though previously he may have been a perfectly honest man, is, says Dr. Baker, obviously lost to all sense of having done wrong. He remains cool when caught, has generally some silly excuse, and regards his position with perfect unconcern. He will appropriate without discrimination articles both useful and worthless. He does not as a rule acquire in the sense that he wishes to retain, for, if he steals freely he bestows generously. Thus while among general paralytics the sexual offenders are influenced by alcohol which excites the sexual sphere, the thieving propensities of those who commit larceny are regarded as in a great measure due to the dementia which reduces the brain to a condition of childishness and re excites the acquisitive tendencies which formed a prominent feature of the mental character of childhood. Homicide occurred in the melancholic variety of general paralysis, but as the melancholy cases were few, homicidal acts were consequently rare.

Insanity as a Biological Problem.—The increased attention paid in recent years to biological problems and heredity, has made itself felt even in the domain of mental pathology. The question of the hereditary transmission of an insane or of a neuropathic and psychopathic taint, has been keenly discussed by alienists and neurologists. In the *Edinburgh Medical Journal*[3] Dr. John MacPherson, Commissioner in Lunacy for Scotland, draws attention to several questions of great practical importance in connection with insanity and heredity. The neuroses are defined as variations from the normal, in respect of a hyper-excitability of the motor or sensory portions of the nervous system, which renders it susceptible to physical impressions and mental suggestion which do not affect normal persons. These patients also have a defect of metabolism, of the nature of which we are at present ignorant, but of the presence of which we are assured by the abundance of neurasthenic symptoms which they exhibit in childhood or during the pubescent and adolescent periods of life. The neuroses all manifest a tendency to periodicity of recurrence and impulsiveness of outbreak. They are all hereditary to a marked extent, and they tend, if they are not fatal, to subside in middle and old age, or as the vital and sexual forces become weaker. The extraordinary feature as regards their distribution in time and space is that they have no geographical or racial limits, but affect all races of men and have existed at all periods of history. Amongst these neuroses the following are included: (a) *Epilepsy.*—This affection, in its true idiopathic form as well as in its other forms, affects both man and animals. Friedenberger and Fröhner[4] state that it occurs at all ages in animals of both sexes. Hirsch[5] states no affection has been so ubiquitous as epilepsy. It would appear to be uninfluenced by climate, soil, race, habits, or geographical location. "It is of the same type, and generally common, among all the nationalities of Europe, the Moorish population of Algiers, the Mongols of North and South Asia, among Malays, Javanese, natives of Peru, and Indians of Brazil." The frequency of this disease is estimated by Hirsch at 1·5 per 1,000 inhabitants in Central and Southern Europe. (b) *Hysteria.*—Hysteria is a psychosis attended by emotional excitement, by peculiar voluntary and convulsive movements of the limbs and body, and by hypnotic states, during which the subject acts and talks impulsively. The attacks are of short duration and paroxysmal in character. Conditions of trance, catalepsy, and mental confusion amounting to a wild frenzy may supervene. Hysteria exists in all nations and has prevailed at all times. The ancient Hindu writings make mention of it, and in modern times it is prevalent among the peoples of Central Asia, Siberia, Europe, South and South-east Asia, in all parts of the New World, and in the Polynesian Isles. Epidemics of hysteria and dancing mania have occurred among many nations in Europe during past centuries.[6] (c) *Alcoholic craving.*—This is a morbid variation characterised by intense craving for the stimulus of drugs, especially alcohol, which some patients exhibit. It is strongly hereditary, commences usually at the adolescent period of life, the symptoms of craving are impulsive and powerful, it occurs often in association with insanity and other neuroses, and its distribution throughout the races of mankind is universal. Legrain states: "We find all forms of insanity present in the ancestors of drinkers—mental defect, hysteria, epilepsy, mania, melancholia, periodic insanity, general paralysis, brain suffering, and apoplexy."[7] The three variations studied above—epilepsy, hysteria, and the alcoholic craving—are, concludes Dr. MacPherson, constant in their existence, *i.e.*, they occur with more or less regularity among the different races of men. They show a universal distribution throughout mankind and, so far as that is possible, among the higher animals. They are hereditary. There remain, therefore, only three explanations of their occurrence, viz, (1) they are fortuitous and due to coincidence; (2) they are caused by the action of the environment; or (3) they are due to variation originating *de novo* with the germ-cells. Dr. MacPherson thinks they cannot be due to accident or fortuitous occurrence; he also rules out of court the action of the environment, saying: "The diseases and defects dealt with have no racial or geographical limit, and, as the environment is universal, this explanation (of their being caused by the environment) may also be set aside." Thus by the method of exclusion he arrives at "genetic variation"—the variation occurring in the germ- and sperm-cells of the human being—as the cause of the neuroses in question. "Genetic variation," says MacPherson, accounts for mental defect and neuroses as it does for discrepancies in stature and in mental ability." Referring to the question of the bodily and anatomical "stigmata of degeneracy" exhibited by the insane and by the neurotics, Dr. MacPherson says: "These are the outward and visible signs of the nervous imperfection or blemishes within. It is acknowledged on all hands that the more grave the incidence of the mental defect, the more numerous and the more pronounced are the physical malformations. Thus, as we pass up the scale from monsters to idiots, imbeciles, and the higher classes of the latter, we find a gradual diminution of the number of bodily malformations, quantitative and qualitative, until they disappear altogether as we emerge upon the apparent normal platform of the race. Besides physical stigmata, there are mental stigmata manifested in the form of epilepsy, hysteria, chorea, tics, and obsessions. The mental defects, like the physical defects, may be due to (*a*) an intrinsic defect of the germ-cells or the embryo, or (*b*) extraneous causes acting on the *fœtus in utero* (infectious) or in infancy. Dr. MacPherson illustrates from the study of dwarfs, who are examples of mal-development met with in every race, that affections of the germ-plasma must be the underlying cause, but that being pathological products dwarfs are infertile and therefore incapable of continuing their race by propagation. Absence of one or more fingers (retrodactylism), polydactylism, and supernumerary toes are regarded as changes of germinal origin. "It is rare to see a diminution of fingers in a non-monstrous individual," said Geoffrey St. Hilaire. Varieties and anomalies of the breasts are also specified by Dr. MacPherson as of similar origin. Albinism is another, and the evidence of Prosper-Lucas and Darwin is said to be to the effect that albinism is "strongly inherited." In true albinos the formation of pigment is arrested in fœtal life. Correlated anomalies co-exist; thus Darwin states that albino cats are also at the same time deaf. Idiocy and albinism often co-exist, and Dr. Felkin of Uganda states he has seen many such cases, a fact which goes to prove the intimate association of cerebral and somatic defect.

[2] Journal of Mental Sci., July 1904. [3] Edin. Med. Jour., June and July 1904. [4] Pathol. and Therap. of Domestic Animals, p. 138. [5] Handbook of Geog. and Historic Pathol., vol. iii., p. 535. [6] Hecker's Epidemics of the Middle Ages. [7] Tuke's Dict. of Psycholog. Med., *sub voce* "Alcoholism."

